# Rates of School Absences in Pediatric Scoliosis Patients and Work Absences in Their Parents/Caregivers: A Retrospective Analysis

**DOI:** 10.3390/jcm13247859

**Published:** 2024-12-23

**Authors:** Ria Paradkar, Christina Regan, Charles P. Nolte, Anthony Stans, William Shaughnessy, Kellen Mulford, Todd A. Milbrandt, A. Noelle Larson

**Affiliations:** Department of Orthopedic Surgery, Mayo Clinic, Rochester, MN 55905, USA; paradkar.ria@mayo.edu (R.P.); regan.christina@mayo.edu (C.R.); charles.p.nolte@gmail.com (C.P.N.J.); stans.anthony@mayo.edu (A.S.); shaughnessy.william@mayo.edu (W.S.); mulford.kellen@mayo.edu (K.M.); milbrandt.todd@mayo.edu (T.A.M.)

**Keywords:** scoliosis, absenteeism, fusion, nonfusion, halo gravity traction, missed school, missed work

## Abstract

**Background/Objectives**: The burden of scoliosis care extends beyond treatment costs and includes missed school for patients and lost income for parents. Chronic absenteeism, defined as more than 18 days of missed school, can have a significant impact on a child’s educational progression, but missed school and work due to scoliosis treatment are not well quantified in the literature. This study investigates absenteeism among scoliosis patients and their caregivers. **Methods**: We conducted a retrospective comparative study of survey results based on surgery timing and surgery type. Patients and caregivers presenting for clinic visits for scoliosis treatment at a single large tertiary care center from 2014 to 2022 were queried. **Results:** We collected 2772 surveys from 1104 unique patients. Of these, 223 surveys from 132 patients were within one year of surgery: 140 post-fusion surveys, 71 post-nonfusion surveys, and 11 post-halo/multistage surgery surveys. A total of 2280 surveys were from 1022 nonoperatively treated patients. School absenteeism was significantly higher for surgeries during the school year compared to summer in both the fusion and nonfusion groups, though work absenteeism showed no significant differences. Halo/multistage surgery patients had the highest rates of absenteeism. **Conclusions**: This study highlights the impacts of scoliosis surgery timing and type on absenteeism among patients and their caregivers. Surgery during summer breaks reduces school absenteeism and academic disruption. Halo/multistage surgery patients face the greatest risk of chronic absenteeism from school, indicating a need for targeted interventions. Optimized surgical timing and planning can help families navigate the educational and financial challenges of scoliosis treatment.

## 1. Introduction

Chronic absenteeism, defined as missing 10% or around 18 days of the school year, is a growing concern in pediatric healthcare [1]. Chronic absenteeism can significantly impact students’ academic and social progress [1]. This problem can arise as a result of multiple factors including socioeconomic barriers, mental health disorders, and medical conditions [2]. Chronic health conditions such as diabetes, asthma, and gastrointestinal diseases have been shown to contribute to increased time away from school [3,4,5]. The association between chronic illness and school absenteeism highlights a pressing need to understand how these conditions may hinder students’ academic and social development.

Chronic absenteeism can have both immediate negative impacts on the child as well as long-term effects. Children who frequently miss school have been shown to have lower test scores and grades as students struggle to keep up with coursework and maintain social connections with peers [6,7,8]. In addition to the detrimental effects on academic performance, chronic absenteeism has also been associated with increased rates of substance use disorders and mental health problems [1,9,10,11]. In addition, a study by Balfanz et al. showed that chronic absenteeism as early as sixth grade can be used to predict which children will not graduate from high school [12]. Despite the breadth of literature regarding absenteeism in many chronic illnesses, it has not yet been studied in adolescents diagnosed with scoliosis. Scoliosis entails both physical and psychosocial effects, making it essential to assess whether treatment of this condition similarly contributes to high rates of absenteeism [13]. It is unclear what the discrete implications of missed school are on patients with scoliosis. However, the cited literature supports the gravity of missed school on children with other medical conditions and the long-term deleterious effects of chronic absenteeism.

The burden of chronic disease extends beyond the child and affects the entire family unit. In addition to the financial burden caused by medical costs, missed days of work by a caregiver may cause increased financial stress as well as a loss of productivity in the workforce [14,15,16]. Scoliosis treatment often requires long-term management with regular checkups, physical therapy sessions, or surgical procedures where caregivers are forced to balance work obligations with the child’s healthcare needs. While the impact of scoliosis has not previously been documented, the effect of other pediatric conditions on caregivers’ missed days of work has been studied in other chronic diseases including traumatic brain injuries (TBI) and juvenile idiopathic arthritis (JIA) [17,18].

The idea that undergoing complex medical procedures during the summer months decreases the number of missed days for the student is a common belief. Marrache et al. has even shown that the number of spinal arthrodesis procedures increases drastically in the months of June, July, and December [19]. During the preoperative period of management, planning interventions to occur during the “less-busy” periods of their years is often a topic of conversation. It is not uncommon to schedule surgeries around the patient’s calendar, after a sports season or a family vacation. Recovery after surgery for scoliosis can be intensive. Many patients require an initial 3-to-5-day hospital stay [20,21,22,23]. After returning home, recovery time can range from 3 to 6 weeks before the patient is able to return to school [24]. Willimon et al. found that patients recovering from a fusion missed an average of 42.3 days of school [24]. Thus, physicians and families are met with a dilemma: postpone surgery and risk further scoliotic progression or schedule an earlier surgical date and risk hindering the child’s educational development.

This study aims to investigate chronic absenteeism in scoliosis patients, examining its impact on academic and work life for both patients and parents. By identifying specific factors including timing of surgery and treatment modality, this study seeks to provide insights for healthcare providers to better support scoliosis patients and their caregivers with the goal of reducing the educational, social, and economic toll of scoliosis care.

## 2. Materials and Methods

This study was a single-center retrospective comparative study. Institutional review board approval was obtained prior to initiation for all aspects of this chart review study (16-008212), which was carried out according to the principles of General Data Protection Regulation. Our IRB noted that approval for this study was carried out through a HIPAA waiver.

As part of the standard of care intake procedure, patients and caregivers presenting for in-person clinic visits for scoliosis treatment at a single large tertiary care center from 2014 to 2022 were queried regarding missed days of school and work. Patients were included who had a spine X-ray ordered for the visit. This included patients with syndromic, congenital, neuromuscular, and idiopathic scoliosis. Patients and caregivers completed the surveys together.

For patients who had undergone surgery, only surveys conducted within one year after the patient’s surgery date were included. Surveys were excluded if they were incomplete or if it had been more than one year since the patient received surgical treatment for scoliosis. For nonoperative patients, all surveys were included.

The survey was developed as part of standard clinical care to understand the burden of scoliosis care on patients and families. Surveys were conducted in English only and administered on a tablet during in-person clinic visits. Although this survey did not undergo formal testing for validation, there have been no reports of confusion or misunderstanding from patients. Patients and caregivers completed the surveys together to ensure that both perspectives were captured. Patients and caregivers were asked (1) whether they missed school or work due to scoliosis care and (2) to estimate the number of missed days in the past year. Survey questions are provided in Table A1 within the Appendix A.

In this survey, patients and caregivers were asked whether they missed school or work and to quantify the number of days missed in the past year due to scoliosis care.

Surveys were analyzed based on two criteria. All surveys included in the analysis of the surgical cohort were conducted within one year of the patient’s surgery date.

1. Surgery Timing: To assess the impact of surgery timing on school absenteeism, patients were categorized based on whether their surgery occurred during the school year (September to May) or during summer break (June to August). For each group, the mean and standard deviation of missed school and work days were calculated. T-tests were conducted with statistical significance set at *p* < 0.05.
2. Surgery Type: Surveys of missed days of work and school were also analyzed across the following treatment groups: fusion, nonfusion, halo/multistage, and nonoperative. Nonfusion interventions included vertebral body tethering and growing rod surgeries. A deep learning-based pediatric spine radiograph classifier was used to determine the type of surgery each patient had undergone [25]. Surgery type was also manually confirmed following classification. Descriptive statistics, including mean and standard deviation, were reported for each treatment group and T-tests were conducted to determine if there were statistically significant differences between groups with significance set at *p* < 0.05. A post hoc power analysis was performed to ensure adequate sample size for missed work in parents/caregivers who underwent fusion and nonfusion surgeries in the summer vs. during the school year.
  All analyses were performed using Microsoft Excel for Microsoft 365 (Microsoft Corp., Redmond, WA, USA).

## 3. Results

A total of 2772 surveys were collected from 1104 unique patients. Of these, 223 surveys from 132 patients were conducted within one year of surgical treatment. A total of 2280 surveys were from 1022 unique patients who were treated without surgery.

Data were analyzed to evaluate school and work absenteeism based on timing of surgery and type of surgery. Patients who were treated by fusion, nonfusion, or halo/multistage surgeries were divided into two groups: those who underwent surgery during the school year (September to May) and those who had surgery during the summer (June to August). Table 1 summarizes findings regarding school and work absenteeism based on both timing of surgery and type of surgery.

### 3.1. Impact of Surgery Timing on School Absenteeism

Among patients who underwent fusion surgery during the summer, 25% of surveys indicated that the patient missed one or more days of school, with an average of 12.3 missed days (SD = 14.2). Nonfusion patients reported a similar rate of absenteeism, with 25% missing school and an average of 5.8 missed days (SD = 5.5). In contrast, 100% of surveys from patients who underwent halo/multistage surgery during the summer reported missing school, with an average of 72.3 days of school (SD = 47.9).

Surgeries performed during the school year were associated with higher rates of school absenteeism for fusion and nonfusion patients. A total of 42.1% of school year fusion surveys reported missing school with an average of 22.0 missed days (SD = 16.9), and 27.5% of nonfusion patient surveys missed school with an average of 19.5 days missed (SD = 12.5). Among halo/multistage patient surveys, 25% reported missing school with an average of 55.0 days (SD = 7.1).

**Figure 1:**
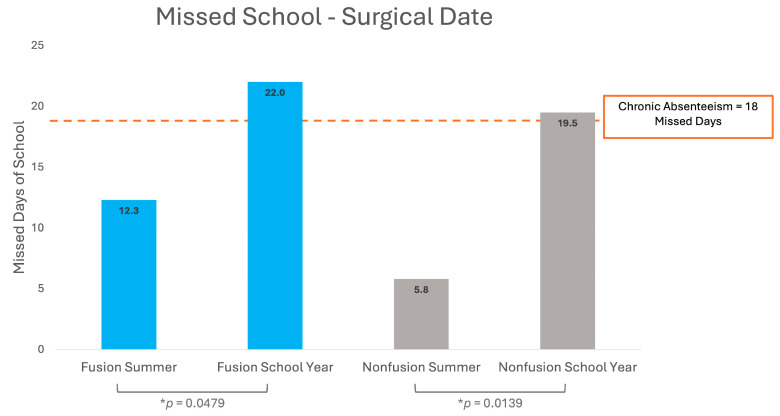
School day absences for pediatric scoliosis surgery patients. * *p* indicates statistical significance (*p* < 0.05).

Figure 1 demonstrates that school year fusion surgeries had a significantly higher number of missed school days compared to summer fusion surgeries (*p* = 0.0479). A similar trend was observed in nonfusion surgeries, with school year procedures resulting in significantly more missed days compared to summer surgeries (*p* = 0.0139).

### 3.2. Impact of Surgery Timing on Work Absenteeism

In the summer fusion surgery group, 59.4% of survey respondents reported missed work, with an average of 16.5 days (SD = 21.7). Nonfusion surgeries had a comparable work absenteeism, with 60.0% of patients missing work for an average of 15.7 days (SD = 14.5). The halo/multistage group had the highest work absenteeism rate, with 100% of surveys indicating missed work and an average of 71.0 days (SD = 50.2).

Fusion surgeries conducted during the school year resulted in the highest work absenteeism rate, with 80.3% reporting missed work days and an average of 17.3 days (SD = 15.5). For nonfusion patients who underwent surgery during the school year, 66.7% of surveys reported missing work, averaging 15.3 days (SD = 11.8). Halo/multistage surgeries showed substantial work absenteeism, with 100% of surveys reporting missed work and an average of 44.9 missed work days (SD = 17.9).

**Figure 2:**
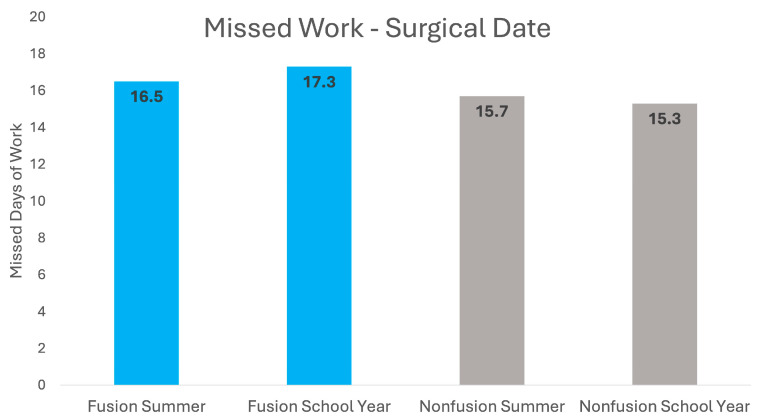
Work absences for parents/caregivers of pediatric scoliosis surgery patients.

Figure 2 compares work absenteeism in fusion and nonfusion surgeries during the summer and school year. In contrast to school absenteeism, there were no significant differences in work absenteeism between summer and school year surgeries for both fusion and nonfusion groups. Overall, parents/caregivers missed 16.6 days of work for a summer surgery compared to 16.7 days of work for a surgery during the school year.

Absenteeism data was further analyzed by type of surgical intervention patients received. Table 2 shows data on missed school and work across four treatment groups: fusion, nonfusion, halo/multistage, and nonoperative.

### 3.3. School Absenteeism by Surgery Type

**Figure 3:**
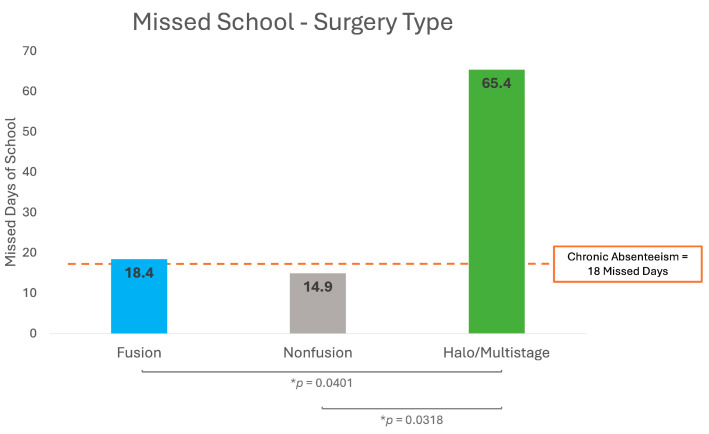
School day absences for pediatric scoliosis surgery patients based on surgery type. * *p* indicates statistical significance (*p* < 0.05).

Distinct patterns of school absenteeism were observed across different surgical interventions. Among 140 surveys collected from 90 patients within one year of fusion surgery, 35.0% of surveys indicated missing school, with an average of 18.4 missed days (SD = 16.7). From 71 surveys collected from 39 patients within one year of nonfusion surgery, 26.8% of surveys reported missed school, with an average of 14.9 days (SD = 12.8). In contrast, 11 surveys from 5 patients who underwent halo/multistage surgery demonstrated the highest rate of absenteeism, with 45.5% indicating missed school and an average of 65.4 missed days (SD = 35.4). Figure 3 shows that halo/multistage surgeries resulted in significantly more missed school days than both fusion (*p* = 0.0401) and nonfusion (*p* = 0.0318) surgeries.

Among 2280 surveys from 1022 nonoperative patients, school absenteeism was the lowest of all groups, with only 24.52% indicating missed school and an average of 9.8 days (SD = 16.2).

### 3.4. Work Absenteeism by Surgery Type

**Figure 4:**
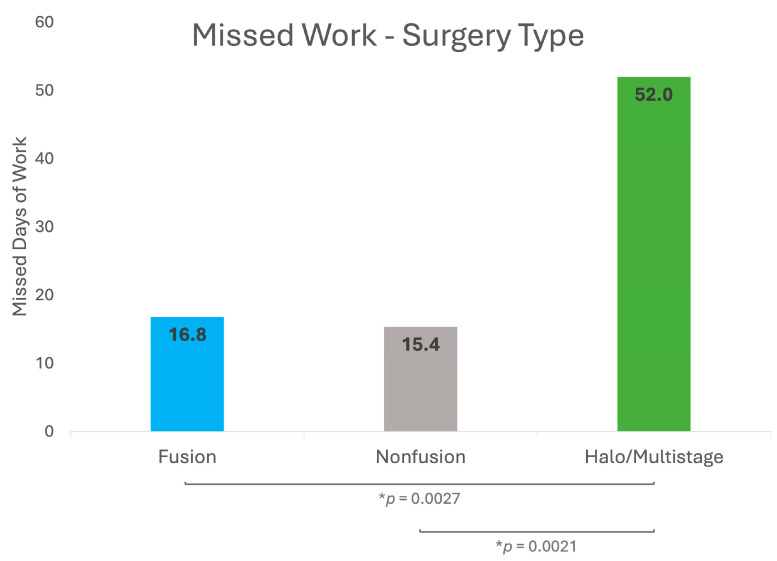
Work absences for parents/caregivers of pediatric scoliosis surgery patients based on surgery type. * *p* indicates statistical significance (*p* < 0.05).

Data on work absenteeism in different treatment groups showed similar patterns. A total of 71.4% of surveys from fusion patients reported missed work, with an average of 16.8 days (SD = 18.0). Among nonfusion patients, 64.8% reported missed work with an average of 15.4 days (SD = 12.4). For halo/multistage patients, 100% of surveys reported missed work with an average of 52.0 days (SD = 29.6). Figure 4 shows that work absenteeism was significantly higher in the halo/multistage group compared to both fusion (*p* = 0.0027) and nonfusion (*p* = 0.0021) groups.

## 4. Discussion

The purpose of this study was to assess the impact of surgery timing and surgery type on school and work absenteeism in pediatric scoliosis patients and their caregivers. By analyzing survey data from patient surveys, we identified absenteeism patterns that can inform surgical planning and post-operative support for patients and their families. Results indicate that both timing and type of surgery significantly influence absenteeism rates. Delineating these associations may help optimize patient care by minimizing academic disruptions and time off work. To our knowledge, this is the first paper to show what is commonly assumed—scoliosis surgery in the summer is better for children’s academic program. In this way, the parents serve as a comparative group, in that they are missing the same amount of work regardless of the season, whereas a child would be expected to miss less school if surgery is performed in the summer. Patient-centered research addresses questions which are of importance to children and caregivers. Thus, this is an important paper to address a common concern for families planning surgical treatment for their child.

### 4.1. Absenteeism Based on Surgery Timing

Results showed that for fusion and nonfusion surgery, procedures performed during the school year were associated with significantly higher rates of absenteeism compared to those conducted during summer months. These findings suggest that undergoing surgery during the academic year places additional strain on students due to concurrent academic responsibilities and medical needs. In contrast, surgeries completed over summer break may allow students greater time to recover without falling behind in school. While halo/multistage surgeries resulted in the highest average days of school missed in both summer and school year procedure groups, they were excluded from this timing analysis due to the low number of patients and surveys.

Previous studies have shown similar patterns of school absenteeism when comparing summer and school year elective surgeries. A study on school-aged athletes receiving operative treatment for knee ligament injuries found that patients who underwent the procedure during the school year reported great academic difficulties compared to those who had their surgery over the holidays or summer break [26]. These results underscore the academic benefits of delaying surgery to school breaks. However, it is important for surgeons and families to weigh the risks of curve progression against the benefit of decreased academic disruption. Delay in surgery can result in increased surgical difficulty, blood loss, complications, or, in the case of vertebral body tethering, a need for fusion surgery should the curve progress past 65 degrees [27,28].

Interestingly, work absenteeism showed no significant differences between summer and school year procedures for both fusion and nonfusion groups. This is likely due to most work obligations remaining constant throughout the year unlike school schedules.

### 4.2. Absenteeism Based on Surgery Type

Survey results analyzed by surgery type revealed that halo/multistage surgeries result in the highest absenteeism for both school and work. Patients who underwent halo/multistage procedures missed an average of 65.4 school days (SD = 35.4), which was significantly more than those who had fusion or nonfusion surgeries. Work absenteeism was similarly elevated, with halo/multistage patients missing an average of 52.0 work days, which was also significantly higher than both the fusion and nonfusion groups.

Halo-gravity traction is an intensive pre-operative intervention for severe spinal deformities [29]. Patients typically remain hospitalized for weeks or months prior to surgery to achieve gradual correction of the deformity. This raises significant challenges for patients undergoing halo-gravity traction, including increased healthcare expenditure and prolonged periods of time away from daily routines for both patients and caregivers [30]. Although outpatient halo-gravity traction has been described, it is not routinely utilized at our center. We did not include specific questions regarding patients who participated in home schooling, online schooling, or other inpatient school mechanisms as part of this dataset.

Conversely, patients undergoing nonoperative scoliosis treatment reported the lowest absenteeism rates for both school and work, with only 24.5% missing school and an average of 9.8 days missed (SD = 16.2), suggesting that non-surgical management is less disruptive to academic attendance. However, it is still more than one might anticipate for patients undergoing bracing or observation for the treatment of scoliosis.

### 4.3. Clinical Implications

The results of this study highlight several clinical implications for management of patients with scoliosis. By informing patients and caregivers about differential absenteeism patterns, orthopedic providers can better guide families preoperatively. This study shows that planning elective surgeries, particularly fusion and nonfusion procedures, during the summer months can benefit patients by minimizing missed school and aligning surgical recovery with periods of reduced academic load. For patients undergoing halo and multistage surgeries, our findings suggest an even greater need for extensive preoperative planning. These patients are at the greatest risk for chronic school absenteeism, which underscores the need for proactive planning for academic accommodations and educational resources to reduce the impact of the missed school days.

Hospital-based school programs are an important tool for children with prolonged school absences [31]. These programs allow for educational continuity, reducing the likelihood of students falling behind in their academic curriculum. Students at risk of chronic absenteeism, such as those undergoing halo traction, should be identified early, and providers should discuss hospital school program options with students and caregivers during preoperative planning. Establishing a standardized process for enrolling patients into these programs and providing support during transitional periods is vital to better serve the academic needs of these children [32,33,34].

This study also calls attention to the substantial amount of work absenteeism associated with scoliosis care for caregivers. Frequent or extended time off from work can have significant social and financial consequences for families, including placing strain on family income, impacting job security, and adding additional financial stress. This is especially true for patients and families with lower socioeconomic status or limited job flexibility. The social, economic, and financial implications of caring for a child with a chronic disease can be intensified by the need for caregivers to take extended time off work. By preparing families for these challenges, surgeons can better support patients and help mitigate the overall burden of treatment on the patient and their family.

### 4.4. Study Limitations

This study has multiple limitations. As with any study which uses survey data, there is potential for reporting errors. Caregivers and patients may not have tracked absences, which could lead to inaccuracies in the reported data. Additionally, only caregivers who were present at each clinic appointment were surveyed. This may mean that in some cases, the surveyed caregiver may not provide a complete story of the family’s employment information. To fully understand the economic impact of scoliosis care, all caregivers should be surveyed. Further, in the interest of respecting the families’ time and to avoid survey fatigue, no data were collected regarding parental occupation. For instance, parents who are teachers may have additional time off to care for their child during the summer. Another limitation was the small sample size of the halo/multistage surgery group. While this group showed the highest absenteeism rates, limited sample sizes restrict the ability to make definitive conclusions. Increasing the sample size for halo/multistage surgeries in the future could provide further insights on the significant academic and occupational impacts for patients and families, respectively. Further, the study could be underpowered to detect a difference between missed days of work in the summer compared to school year surgery. A post hoc power analysis with alpha of 0.05 and a power of 80% showed that over 1000 patients would need to be enrolled to detect a difference between these two cohorts, given the large standard deviation in missed days of work. This study also did not account for factors that could affect postoperative recovery such as comorbidities, complications, or specialized education plans such as, for instance, patients who are home-schooled or who are enrolled in online school. Missed school could also vary by region or country and could also be impacted by patient transportation needs. Future studies should control for such variables by collecting detailed data on the presence of comorbid conditions, educational background/curriculum, complications such as infections or delays in wound healing, etc. Including a matched control group of nonoperatively treated scoliosis patients could allow for a more detailed analysis of the impact of scoliosis treatment on school and work absenteeism.

## 5. Conclusions

In conclusion, this study highlights how scoliosis surgery timing and type influence absenteeism among patients and their caregivers. Delaying surgery to non-academic periods such as summer breaks may be beneficial to students by minimizing missed school days and allowing for fewer academic disruptions. Those undergoing halo gravity traction and multistage surgery are most at risk of chronic absenteeism from school, indicating a critical need for targeted interventions and support for these patients and families. Improved surgical planning can aid families in receiving the necessary resources to navigate educational and financial challenges associated with scoliosis treatment.

## Figures and Tables

**Table 1:** Impact of surgery timing on missed school and work.

Surgery Timing	Surgery Type	# Patients	# Surveys	% Surveys Reported ≥1 Days of Missed School	Average # Missed School Days (SD)	% Surveys Reported ≥1 Days of Missed Work	Average # Missed Work Days (SD)
Summer	Fusion	41	64	25.0	12.3 (14.2)	59.4	16.5 (21.7)
	Nonfusion	11	20	25.0	5.8 (5.5)	60.0	15.7 (14.5)
	Halo/ Multistage	2	3	100.0	72.3 (47.9)	100.0	71 (50.2)
School Year	Fusion	52	76	42.1	22.0 (16.9)	80.3	17.3 (15.5)
	Nonfusion	29	51	27.5	19.5 (12.5)	66.7	15.3 (11.8)
	Halo/ Multistage	3	8	25.0	55.0 (7.1)	100.0	44.9 (17.9)

SD = standard deviation. # = number.

**Table 2:** Impact of surgery type on missed school and work.

	# Patients	# Surveys	% Surveys Reported ≥1 Days of Missed School	Average # Missed School Days (SD)	% Surveys Reported ≥1 Days of Missed Work	Average # Missed Work Days (SD)
Fusion	90	140	35.0	18.4 (16.7)	71.4	16.8 (18.0)
Nonfusion	39	71	26.8	14.9 (12.8)	64.8	15.4 (12.4)
Halo/Multistage	5	11	45.5	65.4 (35.4)	100.0	52.0 (29.6)
Uninstrumented	1022	2280	24.5	9.8 (16.2)	43.0	11.0 (17.1)

SD = standard deviation. # = number.

## Data Availability

Data will be made available upon reasonable request.

## Appendix A

**Table A1:** Missed Days of Work and School Survey.

1. Have you missed any work days in the last year related to your child’s back health/care? (yes/no)
2. How many work days have you missed in the last year related to your child’s back health/care?
3. Has your child missed any school days in the last year related to their back health/care? (yes/no)
4. How many school days has your child missed in the last year related to their back health/care?
